# Andexanet alfa in patients with factor Xa inhibitor-associated intracranial hemorrhage: The prospective observational multicenter ASTRO-DE study

**DOI:** 10.1177/17474930251317385

**Published:** 2025-02-09

**Authors:** Hans-Christoph Diener, Nils Kuklik, Anika Hüsing, Angelika Alonso, Darius G Nabavi, Sven Poli, Maria M Gabriel, Ilko L Maier, Julia Grans

**Affiliations:** 1 Institute for Medical Informatics, Biometry and Epidemiology, Medical Faculty, University of Duisburg-Essen, Essen, Germany; 2 Centre for Clinical Trials Essen, University Hospital Essen, Essen, Germany; 3 Department of Neurology and Mannheim Centre for Translational Neurosciences, Medical Faculty Mannheim, University of Heidelberg, Mannheim, Germany; 4 Department of Neurology, Vivantes Klinikum Neukölln, Berlin, Germany; 5 Department of Neurology and Stroke, University of Tübingen, Tübingen, Germany; 6 Hertie Institute for Clinical Brain Research, University of Tübingen, Tübingen, Germany; 7 Department of Neurology, Hannover Medical School, Hannover, Germany; 8 Department of Neurology, University Medical Center Göttingen, Göttingen, Germany

**Keywords:** Intracranial hemorrhage, anticoagulation, apixaban, rivaroxaban, andexanet alfa, hemostasis

## Abstract

**Background:**

Hematoma expansion after intracranial hemorrhage (ICH) in anticoagulated patients significantly influences clinical outcomes and mortality, emphasizing the need for effective reversal agents. Andexanet alfa is a specific reversal agent for factor Xa-associated major bleeding.

**Aims:**

The Andexanet alfa: non-interventional study at STROke centers in Germany (Deutschland, DE) (ASTRO-DE) study collected real-world evidence on the effect of andexanet alfa on mitigating hematoma expansion and altering prognosis in rivaroxaban- or apixaban-treated patients with ICH.

**Methods:**

ASTRO-DE was a prospective non-interventional cohort study conducted at 25 certified stroke centers in Germany. The primary outcome was the hematoma volume change and the proportion of patients with hematoma growth ⩽33% within 12–72 h or until first control imaging. Secondary endpoints included in-hospital thromboembolic events and mortality up to 90 days.

**Results:**

A total of 137 patients (47.4% male, mean age = 80.0 years) with ICH (92.6% spontaneous, 87.4% intracerebral), mean National Institutes of Health Stroke Scale (NIHSS) on admission of 11.2 points, and mean initial hematoma volume of 26.5 mL (median = 14.1 mL) were analyzed. Ninety patients (65.7%) suffered ICH while treated with apixaban and 47 (34.3%) with rivaroxaban. The median time between symptom onset and application of andexanet alfa was 3.3 h, door-to-needle time was 1.1 h. The mean change in hematoma volume until the first control imaging, conducted after a median of 15.6 h, was 2.3 mL (95% confidence interval (CI) = 0.4–4.2), while the change within 12–72 h was 1.8 mL (95% CI = 0.4–3.2). Hematoma growth ⩽33% was achieved in 90.3% of the 93 evaluable patients based on first control imaging and in 90.5% of the 63 evaluable patients, considering only imaging performed within the 12–72 h window. During hospitalization, death occurred in 30/137 patients (21.9%) and 17 thromboembolic events in 11/137 (8.0%) patients. The 90-day mortality was 47/128 (36.7%).

**Conclusion:**

ASTRO-DE is the first prospective observational study systematically collecting standardized clinical routine data with andexanet alfa treatment. The study demonstrated favorable hemostasis and minimal mean hematoma volume growth in patients with ICH associated with apixaban or rivaroxaban treatment.

**Data access statement:**

Data are available upon reasonable request by contacting the corresponding author.

## Graphical abstract

**Graphical abstract:**
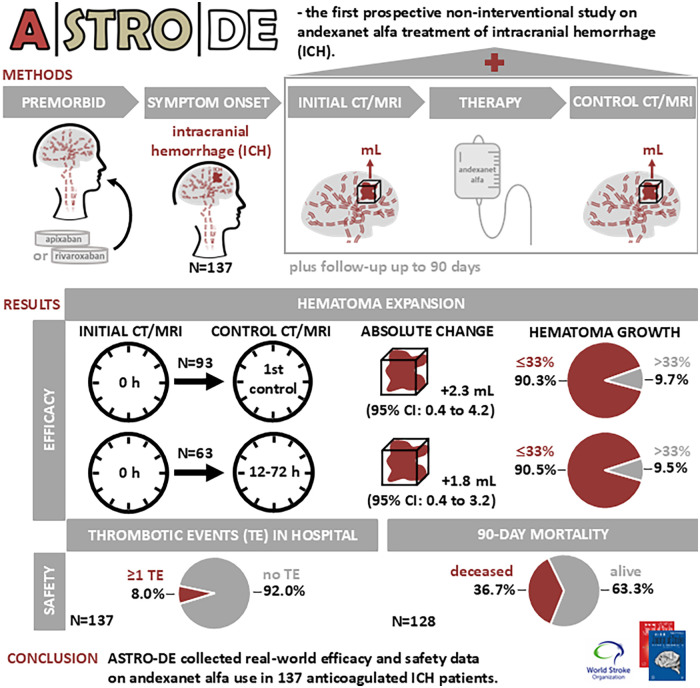

## Introduction

Patients with atrial fibrillation are at high risk of ischemic stroke. Direct oral anticoagulants (DOACs), including factor Xa (FXa) inhibitors such as apixaban, edoxaban, and rivaroxaban, offer therapeutic options for stroke prevention in patients with atrial fibrillation as well as for the prevention and treatment of deep vein thrombosis and pulmonary embolism.^1^ A rare but serious complication of oral anticoagulation, including DOACs, is the occurrence of intracranial hemorrhage (ICH), with a mortality of 30–50% within 90 days.^2,3^ The main modifiable risk factor for poor outcome is hematoma expansion with an incidence of approximately 30% in patients not on anticoagulation and 40% in patients on anticoagulation.^4,5^ Andexanet alfa is a recombinant modified human FXa decoy protein that has the ability to bind and sequester FXa inhibitors, thereby reversing the inhibition of native FXa.^6,7^

To date, several studies have investigated the efficacy and safety of andexanet alfa. Two phase III studies, one with apixaban (ANNEXA-A) and the other one with rivaroxaban (ANNEXA-R), confirmed the efficacy of high- and low-dose regimens of andexanet alfa.^6,7^ ANNEXA-4, an open trial, showed that almost 80% of patients with major hemorrhage achieved hemostatic efficacy.^8^ In the randomized ANNEXA-I trial including patients with ICH treated with FXa inhibitors, andexanet alfa resulted in better control of hematoma expansion than usual care (76.7% vs. 64.6%) but was associated with thrombotic events (10.3% in the andexanet alfa group vs. 5.6% in the usual care group), for example, ischemic strokes.^9^

We conducted the Andexanet alfa: non-interventional study at STROke centers in Germany (Deutschland, DE) (ASTRO-DE), an observational study to evaluate in clinical practice hemostatic efficacy in ICH under treatment with rivaroxaban or apixaban by administering andexanet alfa.

## Methods

### Study design

The ASTRO-DE study was conducted at 25 certified stroke centers in Germany and is a non-interventional study prospectively including patients with symptomatic ICH on effective anticoagulation with rivaroxaban or apixaban treated with andexanet alfa. Recruitment took place from December 2021 to December 2023. There was no simultaneous inclusion of ANNEXA-I patients. The study gathered detailed demographic and clinical data during patients’ hospital stays. The clinical data management system Clincase (Quadratek Data Solutions, Berlin, Germany) was used for standardized data collection. Site investigators estimated intracerebral hematoma volumes using the ABC/2 formula (A: largest diameter in the axial plane, B: largest diameter at an angle of 90° to A in the axial plane, C: number of slices multiplied by the slice thickness).^10^ Subdural, subarachnoid, and intraventricular volumes were measured by the investigators within imaging software. In accordance with the non-interventional study design, no standardized imaging study protocol was used; imaging time and method (computerized cranial tomography or magnetic resonance imaging) were based on the local treating physician’s decision. All patients underwent standardized telephone follow-up after 30 and 90 days to assess disability using the modified Rankin Scale (mRS),^11^ with vital status evaluated after 90 days via queries to residents’ registration offices for those with unsuccessful follow-ups. Serious adverse events (SAEs) occurring from symptom onset until discharge or death were documented using standardized forms and reported within 24 h of awareness. SAE reports included the investigator’s assessment of a possible causal relationship with the administration of andexanet alfa. Data quality was monitored by the Center for Clinical Trials Essen with regular onsite visits and central plausibility assessment.

### Patients

To be included in the study, patients needed to be at least 18 years old, have a symptomatic ICH confirmed by brain imaging, receiving effective anticoagulation treatment with rivaroxaban or apixaban at the time of admission (based on physician’s assessment), and be treated with andexanet alfa. Patients with start of symptoms >24 h before admission were excluded. The measurements of anti-FXa activity were up to the discretion of the investigator.

### Outcomes

The primary endpoint was the absolute change in ICH volume from baseline to first follow-up imaging and, in a more restricted analysis, within 12–72 h. The percentage of patients with hematoma growth ⩽33% and ⩽20% was calculated for both time frames. Patients with follow-up imaging after hematoma evacuation were excluded from the primary analysis. Secondary endpoints included the functional status according to the mRS at discharge, after 30 and 90 days (favorable outcome mRS ⩽ 3; non-favorable outcome mRS = 4–6), intra-hospital mortality and mortality until 7, 30, and 90 days. In addition, the change in stroke severity according to the National Institutes of Health Stroke Scale (NIHSS) 72 h after admission and the incidence and outcomes of in-hospital symptomatic thrombotic events were analyzed.

### Statistical analysis

In previous studies, hematoma expansion defined as >33% relative growth had been reported for approximately one-third of the patients.^12,13^ Al-Shahi et al.^5^ reported >33% relative growth or >6 mL absolute extension in 26% patients not taking anticoagulant therapy and in 40% of patients taking anticoagulant therapy. We therefore recruited 140 patients (120 + 20 excess) to assess the expected proportion of 60–70% patients with hematoma growth ⩽33% within 95% confidence limits below ±9%. Patient characteristics, clinical data, and all endpoints were analyzed exploratively using basic descriptive and inferential statistical methods. Absolute hematoma volume change between initial and control imaging was analyzed using *t*-statistics. Categorical variables were presented in tables showing the number and percentages of observations per category, with the denominator for percentages being the number of patients with non-missing data. Endpoint results were stratified by age, gender, FXa inhibitor, initial hematoma size, initial NIHSS, time since last FXa inhibitor intake, andexanet alfa dose, time interval from first symptoms to initial imaging, bleed cause, localization, and additional therapies. For safety analysis, SAEs were evaluated descriptively, presenting the incidence and proportion of events per patient. Events were coded using MedDRA terminology (version 27.0). In addition, frequency tables for severity, outcomes, and events with potential causal relationships to andexanet alfa were generated. All analyses were conducted using SAS version 9.4 (SAS Institute).

## Results

### Patients

**Figure 1.**
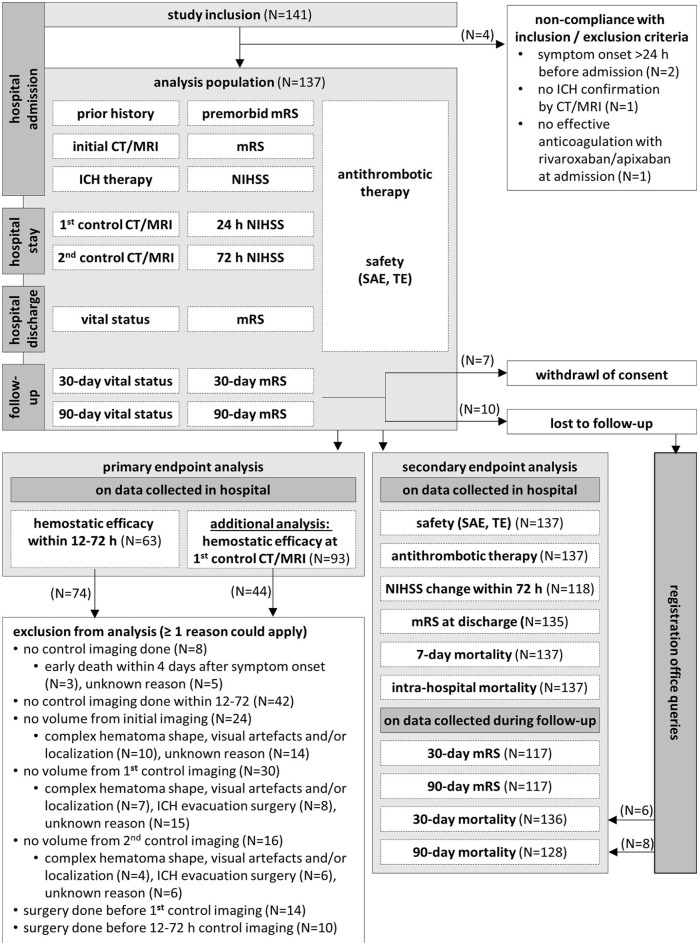
Study inclusions and discontinuations.

**Figure 2.**
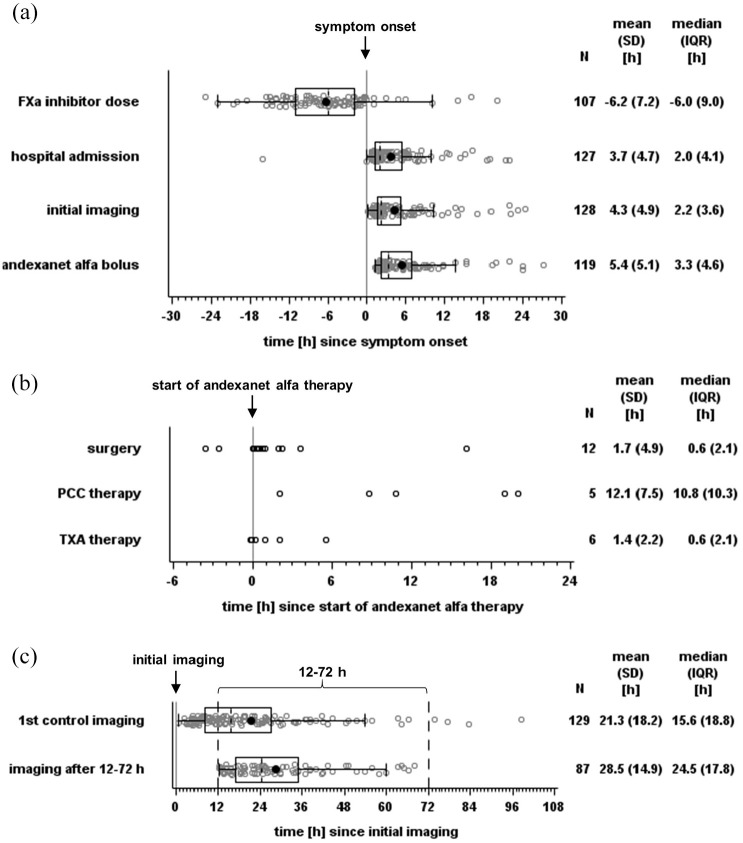
Time until individual treatment steps in hours relative to symptom onset (a), start of andexanet alfa (b), and initial imaging (c) shown as boxplots (box and Tukey whiskers) (a, c) and dotplots (b). FXa: factor Xa; PCC: prothrombin complex concentrate; TXA: tranexamic acid.

A total of 141 patients were enrolled, with four excluded for non-compliance with inclusion criteria, resulting in an analysis population of 137 patients (Figure 1). Demographic and clinical characteristics, including means with standard deviations and medians with interquartile ranges, are presented in Table 1 and Supplementary Table S1. The patients were on average 80 years old and 65 (47.4%) were male. Prior to ICH, 90 (65.7%) patients were treated with apixaban and 47 (34.3%) with rivaroxaban. The median time from the last FXa inhibitor dose to symptom onset was 6.0 h (Figure 2(a)). The most common indication for anticoagulation was atrial fibrillation (121 patients, 88.3%). Andexanet alfa was administered to 85 (62.0%) patients at low doses and to 46 (33.6%) at high doses (other: 6, 4.4%). The majority of bleedings was spontaneous (125 patients, 92.6%) and localized intracerebral (118 patients, 87.4%) (see Table 1 and Supplementary Tables S2–S3). The median time from symptom onset to hospital admission was 2.0 h. Initial imaging and andexanet alfa treatment were performed shortly after hospitalization, with medians of 2.2 h and 3.3 h after symptom onset, respectively, and a median door-to-needle time of 1.1 h (Figure 2(a)). Ten (7.3%) patients received additional reversal therapies: four were treated with prothrombin complex concentrate (PCC; all post-andexanet), five with tranexamic acid (three post-andexanet), and one with both (post-andexanet). ICH was surgically removed in 15 (10.9%) patients, including four who received additional anticoagulant reversal therapy (Table 1 and Supplementary Table S4, Figure 2(b)).

**Table 1.** Baseline characteristics, diagnosis, and therapy.

Characteristics	N	Results
Age (years), median (IQR), mean (SD)	137	82.0 (9.0), 80.0 (8.8)
Gender, no. (%)	137	
Male		65 (47.4%)
Female		72 (52.6%)
Admission unit, no. (%)	137	
Stroke unit		86 (62.8%)
Intensive care unit		35 (25.5%)
Intermediate care unit		5 (3.6%)
Emergency room		11 (8.0%)
Factor Xa inhibitor, no. (%)	137	
Apixaban		90 (65.7%)
Rivaroxaban		47 (34.3%)
Indication for anticoagulation, no. (%)	137	
Atrial fibrillation		121 (88.3%)
Deep vein thrombosis/pulmonary embolism		12 (8.8%)
Renal infarction		2 (1.5%)
Peripheral artery disease		2 (1.5%)
Anti-factor Xa activity (ng/mL), median (IQR), mean (SD)	52	186.2 (139.3), 186.3 (96.4)
Hematoma volume (mL), median (IQR), mean (SD)	113	14.1 (28.9), 26.5 (32.5)
Imaging method, no. (%)	137	
CCT		130 (94.9%)
MRI		7 (5.1%)
Bleed cause, no. (%)	135	
Spontaneous		125 (92.6%)
Trauma-related		10 (7.4%)
Localization, no. (%)	135	
Intracerebral		118 (87.4%)
Subdural		9 (6.7%)
Subarachnoid		8 (5.9%)
Initial NIHSS, median (IQR), mean (SD)	133	9.0 (10.0), 11.2 (8.1)
mRS on admission, median (IQR), mean (SD)	133	5.0 (1.0), 4.2 (1.2)
Andexanet alfa dose, no. (%)	137	
Low (400 mg/15 min + 480 mg/120 min)		85 (62.0%)
High (800 mg/30 min + 960 mg/120 min)		46 (33.6%)
other		6 (4.4%)
Additional therapy, no. (%)	137	
Prothrombin complex concentrate		5 (3.6%)
Tranexamic acid		6 (4.4%)
ICH evacuation surgery		15 (10.9%)

NIHSS: National Institutes of Health Stroke Scale; mRS: modified Rankin Scale.

### Hemostatic efficacy

**Figure 3.**
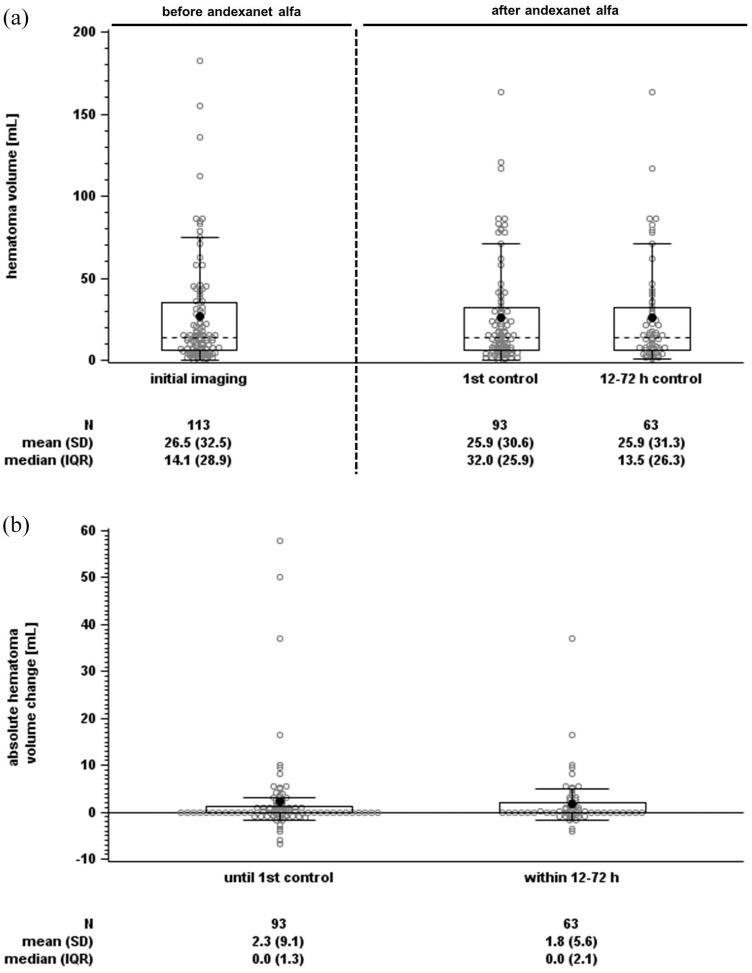
Hematoma volume distribution for individual time points (a) and absolute volume changes (b) shown as boxplots (box and Tukey whiskers).

**Figure 4.**
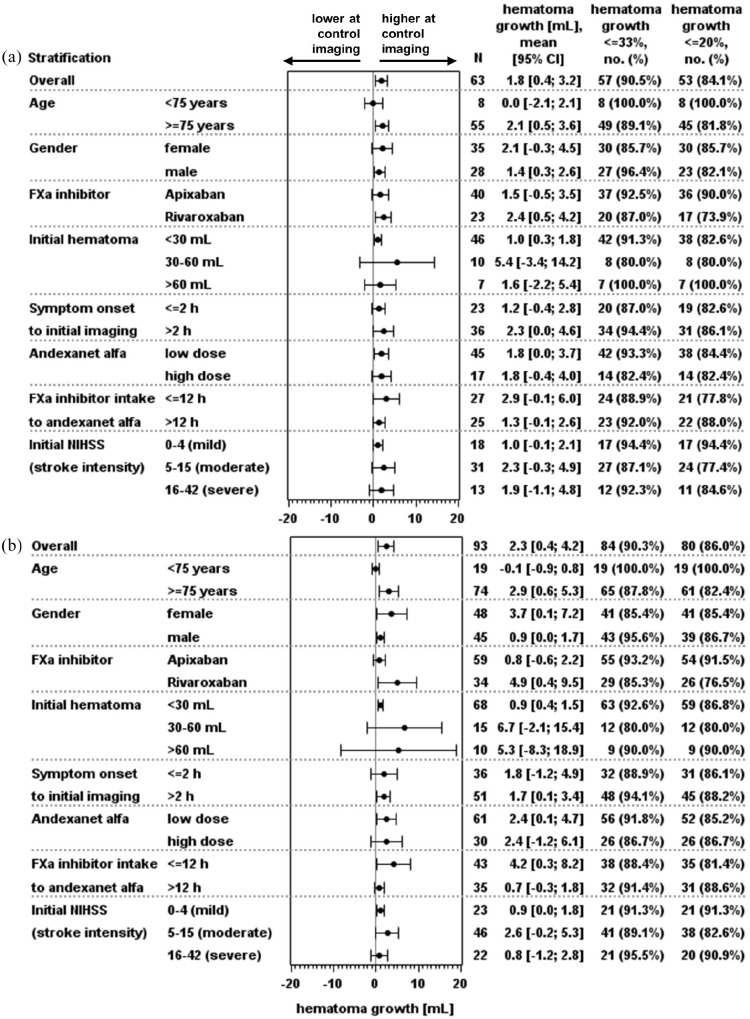
Mean change in hematoma volume within 12–72 h (a) and until first control (b). FXa: factor Xa; NIHSS: National Institutes of Health Stroke Scale.

Mean initial hematoma volume in 113 patients was 26.5 mL, with 14 patients exceeding 60 mL (Table 1 and Figure 3(a)). First control imaging was conducted at a median time of 15.6 h after initial imaging, and 87 control sessions (if possible the first control imaging session, otherwise the deterioration imaging session) were performed after 12–72 h (Figure 2(c)). The primary endpoint assessed hematoma volume change at the first control and within the 12–72 h window, with CT/MRIs of 93 and 63 cases evaluable, respectively (reasons for exclusion: Figure 1). The mean hematoma volume increased minimally, with a mean difference of 2.3 mL (95% confidence interval (CI) = 0.4–4.2 mL) until first control imaging and 1.8 mL (95% CI = 0.4–3.2 mL) within 12–72 h (Figures 3 and 4). Hemostasis assessed through hematoma growth within 12–72 h led to hematoma growth ⩽33% in 90.5% of patients. Similar results were observed when evaluating hemostasis at the first control imaging in all 93 patients (Table 2 and Figure 4). Subgroup analyses further confirmed the consistency of these findings across various factors, including initial hematoma volume, cause of bleeding, localization, and additional therapies (Supplementary Tables S2–S4 and Figure 4 and Supplementary Figure S3).

**Table 2.** Primary and secondary endpoints.

Endpoints	N	Results
Hematoma growth within 12–72 h	63	
Absolute (mL), mean [95% CI]		1.8 [0.4; 3.2]
⩽33%, no. (%)		57 (90.5%)
⩽20%, no. (%)		53 (84.1%)
Hematoma growth until first control	93	
Absolute (mL), mean [95% CI]		2.3 [0.4; 4.2]
⩽33%, no. (%)		84 (90.3%)
⩽20%, no. (%)		80 (86.0%)
Favorable outcome (mRS ⩽ 3) at discharge, no. (%)	135	34 (25.2%)
Favorable outcome (mRS ⩽ 3) at 30 d, no. (%)	117	26 (22.2%)
Favorable outcome (mRS ⩽ 3) at 90 d, no. (%)	117	33 (28.2%)
NIHSS change within 72 h	118	
Absolute, mean [95% CI]		1.5 [0.1; 2.9]
Increase ⩾4 points, no. (%)		26 (22.0%)
Length of hospital stay (days), median (IQR), mean (SD), sum	137	13.0 (12.0), 15.8 (11.2), 2165
Intra-hospital mortality, no. (%)	137	30 (21.9%)
7 days mortality	137	20 (14.6%)
30 days mortality	136	39 (28.7%)
90 days mortality	128	47 (36.7%)
Thrombotic events (TE) in hospital, no. (%)	137	11 (8.0%), 17 TE*
Ischemic stroke		8 (5.8%), 10 TE
Jugular vein thrombosis		1 (0.7%), 1 TE
Myocardial infarction		3 (2.2%), 3 TE*
Peripheral ischemia		1 (0.7%), 1 TE
Peripheral vein thrombosis		1 (0.7%), 1 TE
Sigmoid sinus thrombosis		1 (0.7%), 1 TE
Fatal outcome		4 (2.9%), 7 TE
Restart of any therapeutic anticoagulation		15 (10.9%)
Onset before therapeutic anticoagulation restart		11 (8.0%), 16 TE
Onset after therapeutic anticoagulation restart		1 (0.7%), 1 TE
Onset before therapeutic oral anticoagulation restart		11 (8.0%), 17 TE*
Onset after therapeutic oral anticoagulation restart		0 (0.0%), 0 TE

NIHSS: National Institutes of Health Stroke Scale; mRS: modified Ranking Scale. *One patient suffered a myocardial infarction prior to the administration of andexanet alfa.

### Secondary endpoints

At hospital admission, stroke severity was classified as moderate in most patients with a mean NIHSS score of 11.2 (Table 1 and Supplementary Figure S1). Within the first 72 h, excluding seven patients who died, the NIHSS score worsened by an average of 1.5 points (95% CI = 0.1–2.9). Overall, 22.0% of patients experienced an increase of ⩾4 points in NIHSS within 72 h (Table 2 and Supplementary Figure S1). The premorbid condition prior to ICH according to the mRS was described as unimpaired to moderately impaired in 90.4% of patients (favorable outcome: mRS 0–3), indicating that most patients could walk independently and at most required assistance in everyday life. The mean mRS was 1.5 (median = 1.0). Following ICH, mRS significantly increased and remained high throughout the study. On admission, mRS was, on average, 2.7 points (95% CI = 2.4–2.9) higher than before ICH. At 90-day follow-up, 28.2% of 117 evaluable patients had a favorable outcome with mRS ⩽ 3 (Table 2 and Supplementary Figure S2). During the hospital stay, with a median duration of 13.0 days, 30 patients (21.9%) died. Deaths occurred in 20 (14.6%), 39 (28.7%) and 47 (36.7%) patients within 7, 30, and 90 days, respectively (Table 2). Eleven patients (8.0%) experienced ⩾1 thrombotic event during hospital stay, totaling 17 thrombotic events: ten ischemic strokes, three myocardial infarctions, one jugular vein thrombosis, one sigmoid sinus thrombosis, one peripheral ischemia, and one peripheral vein thrombosis. One myocardial infarction occurred before andexanet alfa administration. Due to the small number of events, potential predictors could not be identified. Sixteen thrombotic events happened before the resumption of any therapeutic antithrombotic therapy; one event occurred after restarting therapeutic parenteral anticoagulation, while no events were reported after resuming oral anticoagulation. The treating physicians assumed a reasonable causal relationship with andexanet alfa in 13 thrombotic events. Four of the patients with thrombotic events died (Table 2 and Supplementary Table S6). Supplementary Tables S5 and S6 provide a complete list of SAEs and summarizing statistics.

## Discussion

In addition to large randomized studies, which are necessary for the approval of a new therapy, it is important to obtain prospective data on the efficacy and safety of a new substance in everyday clinical practice. This was the background for the prospective ASTRO-DE observational study which investigated the efficacy and safety of andexanet alfa in patients who suffered an ICH while on therapy with rivaroxaban or apixaban.

In the ANNEXA-4 study, which included patients with intracranial or gastrointestinal bleeding, the percentage of patients with ICH who achieved hematoma growth ⩽35% within 12 h was 80%.^8^ In the ANNEXA-I study, which compared andexanet alfa and usual therapy in patients with acute ICH, hematoma growth ⩽35% within 12 h was achieved in 165/215 (76.7%) patients.^9^ As an observational study reflecting real-world practice, ASTRO-DE was able to analyze patients who were not included in ANNEXA-I and ANNEXA-4, as these studies had stricter inclusion/exclusion criteria. ANNEXA-4 and ANNEXA-I did not include patients with a hematoma volume >60 mL, resulting in a higher median initial hematoma volume of 14.5 mL in our study compared to ANNEXA-4 (9.5 mL for spontaneous bleedings) and ANNEXA-I (10.5 mL).^9,14^ The door-to-needle time in our study was very short at 1.1 h, compared to >2 h in ANNEXA-4 and ANNEXA-I. Possible reasons included that the measurement of anti-FXa activity in ASTRO-DE was left to the discretion of the physician or hospital as to how they handle it, and the administrative effort for verifying eligibility and inclusion is lower in an observational study. The reduced time to treatment may have positively influenced hemostasis in our study. Another important difference is the time between initial imaging and follow-up imaging, which was set at 12 h in ANNEXA-I and ANNEXA-4.^8,9^ This interval does not correspond to clinical practice in Germany, as indicated by our study, in which first follow-up imaging was performed after a median time of 15.6 h. Moreover, in ASTRO-DE, not all patients received control imaging. We therefore compared the patient characteristics of evaluable and non-evaluable patients. The comparison demonstrates that the primary analysis has the highest representativeness for intracerebral spontaneous bleedings (96.7% intracerebral, 97.8% spontaneous) compared to a higher proportion of subdural, subarachnoid, trauma-related bleedings in the non-evaluable subgroup (Supplementary Table S8 and Figure S3). Baseline parameters such as initial hematoma size, localization, and cause of the bleeding had no impact on the outcome (Figure 4 and Supplementary Table S2 and S3; Supplementary Figure S3).

In ASTRO-DE, 21 (15.3%) patients received additional therapies: 10 (7.3%) received additional reversal therapy and 15 (10.9%) underwent surgery. A subgroup analysis of patients who received additional therapy prior to control imaging compared to those who did not showed no impact on the outcomes (Supplementary Table S4 and Figure S3). This also applies to the comparison of patients with and without additional therapy with regard to secondary endpoints after 30 and 90 days (data not shown).

In comparison to previous studies, our study recorded mortality beyond the 30-day period up to 90 days. The 30-day mortality rate was 28.7% and increased up to 90 days to 36.7%. As mentioned above, due to the less stringent inclusion criteria in our study, we were able to evaluate the secondary endpoints for a larger population. Ninety-day mortality was lower in the subgroup in which the primary endpoint could be evaluated than in the subgroup in which it could not, suggesting that the non-evaluable population included more severely ill patients (Supplementary Table S7). The 30-day mortality in 53 patients with intracranial bleeding in the ARISTOTLE trial on apixaban was 45.3%.^15^ In the ROCKET-AF study, mortality due to intracerebral hemorrhage was 53% (25/47) with rivaroxaban.^16^ A retrospective study reported 30-day mortality after ICH with andexanet alfa treatment of 15.3% (N = 209) versus 48.9% (N = 73) with PCC treatment.^17^ A US hospital-based observational study showed reduced in-hospital mortality in patients with ICH for andexanet alfa (12.6%, N = 666) compared to 4-factor-PCC (23.3%, N = 662).^18^ Mortality within 30 days in the ANNEXA-4 trial was 34/227 (15%), and in the ANNEXA-I trial, it was 73/263 (27.8%) with andexanet alfa and 68/267 (25.5%) in the usual care group.^9,14^ ANNEXA-4 excluded patients with an estimated life expectancy of less than 1 month, which may explain its lower observed mortality compared to ANNEXA-I and ASTRO-DE.^8^ Overall, these studies support that reducing hematoma growth with andexanet alfa reduces mortality. It should be noted, however, that ANNEXA-4, ANNEXA-I, and ASTRO-DE only included specialized stroke centers.

ASTRO-DE also assessed mRS up to 90 days. After symptom onset, the mRS increased from 1.5 (median = 1.0) to 4.2 (median = 5.0) and remained high (4.4 after 90 days, median = 5.0) (Supplementary Figure S2). The NIHSS increase within 72 h after hospital admission was less than 4 points in 92/118 (78.0%) patients. This was expected, as reversal agents cannot reverse initial bleeding damage but reduce hematoma growth risk. In ANNEXA-I, the NIHSS score increased by less than 7 points in 188/214 (87.9%) of participants in 12 h.^9^ In ANNEXA-4, in patients with spontaneous bleeding, who also represent the predominant type of patients included in ASTRO-DE, changes were minimal in the NIHSS within 12 h and the mRS within 30 days.^14^ On admission, the median NIHSS was nine for ANNEXA-I and ASTRO-DE but lower in ANNEXA-4 (six in patients with spontaneous ICH), presumably due to the exclusion of patients a life expectancy under 1 month.^9,14^

Thrombotic events may occur when anticoagulation is discontinued or reversed. In our study, 11 (8.0%) patients had 17 thrombotic events within 26 days, including ten ischemic strokes and three myocardial infarctions. Sixteen thrombotic events occurred before resuming antithrombotic treatment, whereby therapeutic anticoagulation was only resumed in 15 patients in the time window up to discharge. In the ANNEXA-4 and ANNEXA-I study, 21/227 (9.3%) patients and 27/263 (10.3%) patients treated with andexanet alfa suffered thrombotic events, compared to 15/267 (5.6%) usual care ANNEXA-I patients.^9,14^ Whether these thrombotic events are due to andexanet alfa’s procoagulant effect or termination of anticoagulation remains uncertain.^8^

The strength of our observational study is the prospective nature with close monitoring and quality management as well as the systematic and standardized data collection. We had a short door-to-needle time which probably contributed to the good results. The study reflects clinical reality in everyday practice in Germany.

The shortcomings include the fact that the study was not randomized and had no control group. The endpoints, neuroimaging findings, and hematoma sizes were not centrally adjudicated. It was not possible to collect data on the primary outcome in all patients, as some died at an early stage, were operated on, and in others, the repeat CT was either not performed on time or could not be analyzed. FXa levels were not routinely measured on hospital admission.

## Conclusion

ASTRO-DE was the first prospective observational study in 137 patients with intracranial bleeding treated with andexanet alfa while on anticoagulant therapy with rivaroxaban or apixaban and systematically collected standardized clinical routine data. ASTRO-DE showed hematoma growth ⩽33% in 91% of evaluable patients with ICH, and 8.0% of patients suffered at least one thrombotic event during hospitalization.

## Supplemental Material

sj-docx-1-wso-10.1177_17474930251317385 – Supplemental material for Andexanet alfa in patients with factor Xa inhibitor-associated intracranial hemorrhage: The prospective observational multicenter ASTRO-DE study Supplemental material, sj-docx-1-wso-10.1177_17474930251317385 for Andexanet alfa in patients with factor Xa inhibitor-associated intracranial hemorrhage: The prospective observational multicenter ASTRO-DE study by Hans-Christoph Diener, Nils Kuklik, Anika Hüsing, Angelika Alonso, Darius G Nabavi, Sven Poli, Maria M Gabriel, Ilko L Maier and Julia Grans in International Journal of Stroke

## References

[1] Diener HC Ntaios G O’Donnell M Easton JD. Non-vitamin-K oral anticoagulants (NOACs) for the prevention of secondary stroke. Expert Opin Pharmacother 2018; 19: 1597–1602. 30152249 10.1080/14656566.2018.1515913

[2] Lopes RD Guimaraes PO Kolls BJ , et al. Intracranial hemorrhage in patients with atrial fibrillation receiving anticoagulation therapy. Blood 2017; 129: 2980–2987. 28356246 10.1182/blood-2016-08-731638

[3] Apostolaki-Hansson T Ullberg T Norrving B Petersson J. Prognosis for intracerebral hemorrhage during ongoing oral anticoagulant treatment. Acta Neurol Scand 2019; 139: 415–421. 30657164 10.1111/ane.13068

[4] Purrucker JC Haas K Rizos T , et al. Early clinical and radiological course, management, and outcome of intracerebral hemorrhage related to new oral anticoagulants. JAMA Neurol 2016; 73: 169–177. 26660118 10.1001/jamaneurol.2015.3682

[5] Al-Shahi Salman R Frantzias J Lee RJ , et al. Absolute risk and predictors of the growth of acute spontaneous intracerebral haemorrhage: a systematic review and meta-analysis of individual patient data. Lancet Neurol 2018; 17: 885–894. 30120039 10.1016/S1474-4422(18)30253-9 PMC6143589

[6] Connolly SJ Milling TJ Jr Eikelboom JW , et al. Andexanet alfa for acute major bleeding associated with factor Xa inhibitors. N Engl J Med 2016; 375: 1131–1141. 27573206 10.1056/NEJMoa1607887 PMC5568772

[7] Siegal D Lu G Leeds JM , et al. Safety, pharmacokinetics, and reversal of apixaban anticoagulation with andexanet alfa. Blood Adv 2017; 1: 1827–1838. 29296829 10.1182/bloodadvances.2017007112 PMC5728098

[8] Connolly SJ Crowther M Eikelboom JW , et al. Full study report of andexanet alfa for bleeding associated with factor Xa inhibitors. N Engl J Med 2019; 380: 1326–1335. 30730782 10.1056/NEJMoa1814051 PMC6699827

[9] Connolly SJ Sharma M Cohen AT , et al. Andexanet for factor Xa inhibitor-associated acute intracerebral hemorrhage. N Engl J Med 2024; 390: 1745–1755. 38749032 10.1056/NEJMoa2313040

[10] Sims JR Gharai LR Schaefer PW , et al. ABC/2 for rapid clinical estimate of infarct, perfusion, and mismatch volumes. Neurology 2009; 72: 2104–2110. 19528517 10.1212/WNL.0b013e3181aa5329 PMC2697964

[11] Bruno A Akinwuntan AE Lin C , et al. Simplified modified Rankin scale questionnaire: reproducibility over the telephone and validation with quality of life. Stroke 2011; 42: 2276–2279. 21680905 10.1161/STROKEAHA.111.613273

[12] Burchell SR Tang J Zhang JH. Hematoma expansion following intracerebral hemorrhage: mechanisms targeting the coagulation cascade and platelet activation. Curr Drug Targets 2017; 18: 1329–1344. 28378693 10.2174/1389450118666170329152305 PMC6894484

[13] Li Z You M Long C , et al. Hematoma expansion in intracerebral hemorrhage: an update on prediction and treatment. Front Neurol 2020; 11: 702. 32765408 10.3389/fneur.2020.00702 PMC7380105

[14] Demchuk AM Yue P Zotova E , et al. Hemostatic efficacy and Anti-FXa (Factor Xa) reversal with andexanet alfa in intracranial hemorrhage: ANNEXA-4 substudy. Stroke 2021; 52: 2096–2105. 33966491 10.1161/STROKEAHA.120.030565 PMC8140631

[15] Granger CB Alexander JH McMurray JJ , et al. Apixaban versus warfarin in patients with atrial fibrillation. N Engl J Med 2011; 365: 981–992. 21870978 10.1056/NEJMoa1107039

[16] Hankey GJ Stevens SR Piccini JP , et al. Intracranial hemorrhage among patients with atrial fibrillation anticoagulated with warfarin or rivaroxaban: the rivaroxaban once daily, oral, direct factor Xa inhibition compared with vitamin K antagonism for prevention of stroke and embolism trial in atrial fibrillation. Stroke 2014; 45: 1304–1312. 24743444 10.1161/STROKEAHA.113.004506

[17] Cohen AT Lewis M Connor A , et al. Thirty-day mortality with andexanet alfa compared with prothrombin complex concentrate therapy for life-threatening direct oral anticoagulant-related bleeding. J Am Coll Emerg Physicians Open 2022; 3: e12655. 10.1002/emp2.12655 PMC8898077 35280921

[18] Dobesh PP Fermann GJ Christoph MJ , et al. Lower mortality with andexanet alfa vs 4-factor prothrombin complex concentrate for factor Xa inhibitor-related major bleeding in a U.S. Res Pract Thromb Haemost 2023; 7: 102192. 37753225 10.1016/j.rpth.2023.102192 PMC10518480

