# Supplementary material for: Andexanet alfa in patients with factor Xa inhibitor-associated intracranial hemorrhage: The prospective observational multicenter ASTRO-DE study
Source: Int J Stroke. 2025 Feb 9;20(7):831–42. doi: 10.1177/17474930251317385 (PMC12264301; doi:10.1177/17474930251317385)
Supplement: sj-docx-1-wso-10.1177_17474930251317385 – Supplemental material for Andexanet alfa in patients with factor Xa inhibitor-associated intracranial hemorrhage: The prospective observational multicenter ASTRO-DE study [file sj-docx-1-wso-10.1177_17474930251317385.docx]

**Table S1.** Concomitant medication and medical history.

| **Characteristic** | **Analysis** | **N** | **Result** |
| --- | --- | --- | --- |
| Concomitant medication, no (%) | | 137 |  |
| antiplatelet therapy | |  | 10 (7.3%) |
| Acetylsalicylic acid | |  | 7 (5.1%) |
| Clopidogrel | |  | 3 (2.2%) |
| nonsteroidal anti-inflammatory drugs | |  | 3 (2.2%) |
| Diclofenac | |  | 2 (1.5%) |
| Ibuprofen | |  | 1 (0.7%) |
| Medical history, no (%) | | 137 |  |
| atrial fibrillation | |  | 121 (88.3%) |
| cancer / brain metastases | |  | 19 (13.9%) |
| coronary artery disease | |  | 23 (16.8%) |
| deep vein thrombosis | |  | 9 (6.6%) |
| diabetes | |  | 32 (23.4%) |
| heart failure | |  | 27 (19.7%) |
| hyperlipidemia | |  | 39 (28.5%) |
| hypertension | |  | 110 (80.3%) |
| intracranial bleeding | |  | 12 (8.8%) |
| myocardial infarction | |  | 10 (7.3%) |
| peripheral arterial occlusive disease | |  | 9 (6.6%) |
| pulmonary embolism | |  | 9 (6.6%) |
| stroke | |  | 21 (15.3%) |
| transient ischemic attack | |  | 7 (5.1%) |

**Table S2.** Evaluability of different bleed causes and localizations in the primary endpoint.

| **bleed cause** | **localization** | **all patients (N=137),**  **no. (%)** | **primary endpoint analysis:**  **hematoma growth** | |
| --- | --- | --- | --- | --- |
|  |  |  | **within 12-72 h (N=63), no. (%)** | **until 1st control (N=93), no. (%)** |
| spontaneous | intracerebral | 117 (85.4%) | 60 (95.2%) | 89 (95.7%) |
|  | subdural | 6 (4.4%) | 0 (0.0%) | 1 (1.1%) |
|  | subarachnoid | 2 (1.5%) | 0 (0.0%) | 0 (0.0%) |
| traumatic | intracerebral | 1 (0.7%) | 0 (0.0%) | 0 (0.0%) |
|  | subdural | 3 (2.2%) | 0 (0.0%) | 0 (0.0%) |
|  | subarachnoid | 6 (4.4%) | 2 (3.2%) | 2 (2.2%) |
| missing | missing | 2 (1.5%) | 1 (1.6%) | 1 (1.1%) |

Due to rounding, the sum of the percentages may not equal 100.

**Table S3.** Evaluability of different intracerebral localizations in the primary endpoint.

| **intracerebral localization ICD-10 code** | **all intracerebral bleedings (N=118), no. (%)** | **primary endpoint analysis:**  **hematoma growth** | |
| --- | --- | --- | --- |
|  |  | **within 12-72 h (N=60), no. (%)** | **until 1st control (N=89), no. (%)** |
| I61.0 (cerebral hemisphere, subcortical) | 67 (56.8%) | 38 (63.3%) | 57 (64.0%) |
| I61.1 (cerebral hemisphere, cortical) | 16 (13.6%) | 9 (15.0%) | 12 (13.5%) |
| I61.2 (cerebral hemisphere, unspecified) | 9 (7.6%) | 2 (3.3%) | 3 (3.4%) |
| I61.3 (brainstem) | 2 (1.7%) | 1 (1.7%) | 1 (1.1%) |
| I61.4 (cerebellum) | 10 (8.5%) | 3 (5.0%) | 7 (7.9%) |
| I61.5 (intraventricular) | 4 (3.4%) | 2 (3.3%) | 3 (3.4%) |
| I61.0 + I61.1 | 1 (0.9%) | 1 (1.7%) | 1 (1.1%) |
| I61.0 + I61.5 | 4 (3.4%) | 2 (3.3%) | 3 (3.4%) |
| I61.1 + I61.5 | 1 (0.9%) | 1 (1.7%) | 1 (1.1%) |
| I61.2 + I61.5 | 1 (0.9%) | 1 (1.7%) | 1 (1.1%) |
| I61.4 + I61.5 | 1 (0.9%) | 0 (0.0%) | 0 (0.0%) |
| missing* | 2 (1.7%) | 0 (0.0%) | 0 (0.0%) |

Due to rounding, the sum of the percentages may not equal 100. *Including the one traumatic intracerebral hemorrhage.

**Table S4.** Timing of additional therapies in patients with available initial and follow-up volume.

| **additional therapies** | **all patients (N=137),**  **no. (%)** | **Evaluable patients** | | | |
| --- | --- | --- | --- | --- | --- |
|  |  | **hematoma growth**  **within 12-72 h**  **(N=67), no. (%)** | | **hematoma growth**  **until 1st available control (N=99), no. (%)** | |
|  |  | **therapy before control** | **therapy after control**  **or never** | **therapy before control** | **therapy after control**  **or never** |
| none | 116 (84.7%) | - | 59 (88.1%) | - | 87 (87.9%) |
| surgery | 11 (8.0%) | 3 (4.5%)* | 0 (0.0%) | 4 (4.0%)* | 1 (1.0%) |
| PCC | 2 (1.5%) | 1 (1.5%) | 1 (1.5%) | 1 (1.0%) | 1 (1.0%) |
| TXA | 4 (2.9%) | 2 (3.0%) | 0 (0.0%) | 2 (2.0%) | 1 (1.0%) |
| surgery + PCC | 2 (1.5%) | 0 (0.0%) | 0 (0.0%) | 0 (0.0%) | 0 (0.0%) |
| surgery + TXA | 1 (0.7%) | 1 (1.5%)* | 0 (0.0%) | 1 (1.0%)* | 0 (0.0%) |
| PCC + TXA | 0 (0.0%) | 0 (0.0%) | 0 (0.0%) | 0 (0.0%) | 0 (0.0%) |
| surgery + PCC + TXA | 1 (0.7%) | 0 (0.0%) | 0 (0.0%) | 1 (1.0%)* | 0 (0.0.%) |

Due to rounding, the sum of the percentages may not equal 100. PCC: prothrombin complex concentrate; TXA: tranexamic acid. *Evaluable patients who underwent surgery prior to control imaging were excluded from the primary analyses.

**Table S5.** Serious adverse events (SAE) in hospital.

| **Primary System Organ Class (SOC)**  **Preferred Term** | **Patients, no. (%)**  **(N=137)** | **SAE, no. (%)**  **(N=69)** |
| --- | --- | --- |
| **All SAE** | 52 (38.0%) | 69 (100.0%) |
| **Cardiac disorders** | 7 (5.1%) | 7 (10.1%) |
| acute myocardial infarction | 3 (2.2%) | 3 (4.3%) |
| cardiac arrest | 2 (1.5%) | 2 (2.9%) |
| cardiac failure | 2 (1.5%) | 2 (2.9%) |
| **General disorders and administration site conditions** | 23 (16.8%) | 23 (33.3%) |
| complication associated with device | 1 (0.7%) | 1 (1.4%) |
| death* | 22 (16.1%) | 22 (31.9%) |
| **Infections and infestations** | 12 (8.8%) | 12 (17.4%) |
| COVID-19 pneumonia | 1 (0.7%) | 1 (1.4%) |
| pneumonia | 6 (4.4%) | 6 (8.7%) |
| pneumonia aspiration | 2 (1.5%) | 2 (2.9%) |
| prosthetic valve endocarditis | 1 (0.7%) | 1 (1.4%) |
| superinfection bacterial | 1 (0.7%) | 1 (1.4%) |
| tracheobronchitis | 1 (0.7%) | 1 (1.4%) |
| **Investigations** | 1 (0.7%) | 1 (1.4%) |
| international normalized ratio increased | 1 (0.7%) | 1 (1.4%) |
| **Nervous system disorders** | 14 (10.2%) | 18 (26.1%) |
| brain edema | 1 (0.7%) | 1 (1.4%) |
| cerebral hemorrhage | 1 (0.7%) | 1 (1.4%) |
| cerebral ischemia | 2 (1.5%) | 2 (2.9%) |
| embolic stroke | 1 (0.7%) | 1 (1.4%) |
| ischemic stroke | 5 (3.6%) | 7 (10.1%) |
| myoclonic epilepsy | 1 (0.7%) | 1 (1.4%) |
| sigmoid sinus thrombosis | 1 (0.7%) | 1 (1.4%) |
| status epilepticus | 3 (2.2%) | 3 (4.3%) |
| subarachnoid hemorrhage | 1 (0.7%) | 1 (1.4%) |
| **Psychiatric disorders** | 2 (1.5%) | 2 (2.9%) |
| delirium | 2 (1.5%) | 2 (2.9%) |
| **Respiratory, thoracic and mediastinal disorders** | 2 (1.5%) | 2 (2.9%) |
| pneumothorax | 1 (0.7%) | 1 (1.4%) |
| respiratory failure | 1 (0.7%) | 1 (1.4%) |
| **Surgical and medical procedures** | 1 (0.7%) | 1 (1.4%) |
| cardiac pacemaker adjustment | 1 (0.7%) | 1 (1.4%) |
| **Vascular disorders** | 2 (1.5%) | 3 (4.3%) |
| jugular vein thrombosis | 1 (0.7%) | 1 (1.4%) |
| peripheral ischemia | 1 (0.7%) | 1 (1.4%) |
| peripheral vein thrombosis | 1 (0.7%) | 1 (1.4%) |

*Death due to index event.

**Table S6.** Intensity, seriousness, outcome and causal relationship to andexanet alfa of intra-hospital serious adverse events (SAE).

| **Criterion** | **Characteristic** | **Patients, no. (%)**  **(N=137)** | **SAE, no. (%)**  **(N=69)** |  |
| --- | --- | --- | --- | --- |
| All SAE | all SAE | 52 (38.0%) | 69 (100.0%) | |
| Intensity | mild | 3 (2.2%) | 3 (4.3%) | |
|  | moderate | 11 (8.0%) | 13 (18.8%) | |
|  | severe | 41 (29.9%) | 53 (76.8%) | |
| Seriousness | fatal* | 31 (22.6%) | 34 (49.3%) | |
|  | immediately life threatening | 6 (4.4%) | 8 (11.6%) | |
|  | (prolonged) hospitalization | 15 (10.9%) | 17 (24.6%) | |
|  | disabling | 4 (2.9%) | 6 (8.7%) | |
|  | other medical important event | 4 (2.9%) | 4 (5.8%) | |
| Outcome | recovered | 17 (12.4%) | 24 (34.8%) |  |
|  | not yet recovered | 4 (2.9%) | 6 (8.7%) | |
|  | recovered with sequelae | 2 (1.5%) | 2 (2.9%) | |
|  | fatal* | 31 (22.6%) | 34 (49.3%) | |
|  | unknown | 3 (2.2%) | 3 (4.3%) | |
| Reasonable causal relationship to andexanet alfa | yes† | 9 (6.6%) | 13 (18.8%) | |
|  | no | 45 (32.8%) | 54 (78.3%) | |
|  | missing | 2 (1.5%) | 2 (2.9%) | |

*One death occurred after discharge from hospital. †Preferred terms: Acute myocardial infarction, cerebral ischemia, ischemic stroke, jugular vein thrombosis, peripheral vein thrombosis, sigmoid sinus thrombosis, i.e. all SAEs reported as causally related to andexanet alfa were thrombotic events. This corresponds to 13 of the 17 thrombotic events (9 of the 11 patients) listed in Table 2.

**Table S7.** Mortality after 90 days (N=128) in patients considered and not considered in primary endpoint analysis.

| **Population** | **N** | **90-days mortality, no. (%)** |
| --- | --- | --- |
| ASTRO-DE population | 137 | 47/128 (36.7%) |
| Included in primary endpoint analysis on hemostasis within 12-72 h | 63 | 20/59 (33.9%) |
| Excluded from primary endpoint analysis on hemostasis within 12-72 h | 74 | 27/69 (39.1%) |
| Included in primary endpoint analysis on hemostasis until 1st control | 93 | 32/88 (36.4%) |
| Excluded from primary endpoint analysis on hemostasis until 1st control | 44 | 15/40 (37.5%) |

**Table S8.** Comparison of baseline characteristics, diagnosis and treatment in the (non-)analysis populations of the primary endpoint.

| **Characteristic** | **All patients (N=137)** | | **Primary analysis: hematoma growth** | | | | | | | |
| --- | --- | --- | --- | --- | --- | --- | --- | --- | --- | --- |
|  |  |  | **until 1^st^ control** | | | | **within 12-72 h** | | | |
|  |  |  | **Evaluable**  **(N=93)** | | **Non-evaluable (N=44)** | | **Evaluable**  **(N=63)** | | **Non-evaluable**  **(N=74)** | |
|  | **N** | **Result** | **N** | **Result** | **N** | **Result** | **N** | **Result** | **N** | **Result** |
| Age [y], median (IQR), mean (SD) | 137 | 82.0 (9.0), 80.0 (8.8) | 93 | 81.0 (10.0), 80.0 (7.8) | 44 | 82.0 (11.0), 80.1 (10.7) | 63 | 82.0 (8.0), 81.2 (7.1) | 74 | 81.0 (12.0),  79.1 (10.0) |
| Gender, no. (%) | 137 |  | 93 |  | 44 |  | 63 |  | 74 |  |
| male |  | 65 (47.4%) |  | 45 (48.4%) |  | 20 (45.5%) |  | 28 (44.4%) |  | 37 (50.0%) |
| female |  | 72 (52.6%) |  | 48 (51.6%) |  | 24 (54.5%) |  | 35 (55.6%) |  | 37 (50.0%) |
| Admission unit, no. (%) | 137 |  | 93 |  | 44 |  | 63 |  | 74 |  |
| stroke unit |  | 86 (62.8%) |  | 69 (74.2%) |  | 17 (38.6%) |  | 51 (81.0%) |  | 35 (47.3%) |
| intensive care unit |  | 35 (25.5%) |  | 17 (18.3%) |  | 18 (40.9%) |  | 10 (15.9%) |  | 25 (33.8%) |
| intermediate care unit |  | 5 (3.6%) |  | 3 (3.2%) |  | 2 (4.5%) |  | 0 (0.0%) |  | 5 (6.8%) |
| emergency room |  | 11 (8.0%) |  | 4 (4.3%) |  | 7 (15.9%) |  | 2 (3.2%) |  | 9 (12.2%) |
| Factor Xa inhibitor, no. (%) | 137 |  | 93 |  | 44 |  | 63 |  | 74 |  |
| Apixaban |  | 90 (65.7%) |  | 59 (63.4%) |  | 31 (70.5%) |  | 40 (63.5%) |  | 50 (67.6%) |
| Rivaroxaban |  | 47 (34.3%) |  | 34 (36.6%) |  | 13 (29.5%) |  | 23 (36.5%) |  | 24 (32.4%) |
| Indication for anticoagulation, no. (%) | 137 |  | 93 |  | 44 |  | 63 |  | 74 |  |
| atrial fibrillation |  | 121 (88.3%) |  | 83 (89.2%) |  | 38 (86.4%) |  | 58 (92.1%) |  | 63 (85.1%) |
| deep vein thrombosis / pulmonary embolism |  | 12 (8.8%) |  | 6 (6.5%) |  | 6 (13.6%) |  | 3 (4.8%) |  | 9 (12.2%) |
| renal infarction |  | 2 (1.5%) |  | 2 (2.2%) |  | 0 (0.0%) |  | 1 (1.6%) |  | 1 (1.4%) |
| peripheral artery disease |  | 2 (1.5%) |  | 2 (2.2%) |  | 0 (0.0%) |  | 1 (1.6%) |  | 1 (1.4%) |
| Anti-factor Xa activity [ng/mL],  median (IQR), mean (SD) | 52 | 186.2 (139.3), 186.3 (96.4) | 38 | 188.9 (105.0), 192.1 (94.1) | 14 | 117.9 (156.5), 170.7 (104.4) | 27 | 191.0 (120.0),  195.9 (92.7) | 25 | 156.0 (147.0), 176.0 (101.1) |
| Anti-factor Xa activity [IU/mL],  median (IQR), mean (SD) | 8 | 0.8 (0.9), 0.8 (0.6) | 4 | 0.7 (0.9), 0.7 (0.6) | 4 | 0.8 (1.1), 1.0 (0.8) | 2 | 0.8 (1.2),  0.8 (0.8) | 6 | 0.8 (0.7),  0.9 (0.7) |
| Imaging method, no. (%) | 137 |  | 93 |  | 44 |  | 63 |  | 74 |  |
| CCT |  | 130 (94.9%) |  | 88 (94.6%) |  | 42 (95.5%) |  | 61 (96.8%) |  | 69 (93.2%) |
| MRI |  | 7 (5.1%) |  | 5 (5.4%) |  | 2 (4.5%) |  | 2 (3.2%) |  | 5 (6.8%) |
| Hematoma volume [mL],  median (IQR), mean (SD) | 113 | 14.1 (28.9), 26.5 (32.5) | 93 | 14.0 (25.9), 23.6 (27.7) | 20 | 20.6 (43.8), 39.7 (47.6) | 63 | 12.1 (26.3), 24.1 (29.7) | 50 | 15.2 (33.5),  29.5 (35.7) |
| Bleed cause, no. (%) | 135 |  | 92 |  | 43 |  | 62 |  | 73 |  |
| spontaneous |  | 125 (92.6%) |  | 90 (97.8%) |  | 35 (81.4%) |  | 60 (96.8%) |  | 65 (89.0%) |
| trauma-related |  | 10 (7.4%) |  | 2 (2.2%) |  | 8 (18.6%) |  | 2 (3.2%) |  | 8 (11.0%) |
| Localization, no. (%) | 135 |  | 92 |  | 43 |  | 62 |  | 73 |  |
| intracerebral |  | 118 (87.4%) |  | 89 (96.7%) |  | 29 (67.4%) |  | 60 (96.8%) |  | 58 (79.5%) |
| subdural |  | 9 (6.7%) |  | 1 (1.1%) |  | 8 (18.6%) |  | 0 (0.0%) |  | 9 (12.3%) |
| subarachnoid |  | 8 (5.9%) |  | 2 (2.2%) |  | 6 (14.0%) |  | 2 (3.2%) |  | 6 (8.2%) |
| Initial NIHSS,  median (IQR), mean (SD) | 133 | 9.0 (10.0), 11.2 (8.1) | 91 | 10.0 (11.0), 11.1 (7.7) | 42 | 9.0 (11.0), 11.5 (8.9) | 62 | 10.0 (11.0), 10.7 (7.2) | 71 | 9.0 (11.0),  11.6 (8.8) |
| mRS on admission,  median (IQR), mean (SD) | 133 | 5.0 (1.0),  4.2 (1.2) | 91 | 5.0 (1.0),  4.2 (1.2) | 42 | 4.5 (1.0),  4.2 (1.2) | 63 | 5.0 (1.0),  4.2 (1.2) | 70 | 5.0 (1.0),  4.2 (1.2) |
| Andexanet alfa dose, no. (%) | 137 |  | 93 |  | 44 |  | 63 |  | 74 |  |
| low  (400 mg/15 min + 480 mg/120 min) |  | 85 (62.0%) |  | 61 (65.6%) |  | 24 (54.5%) |  | 45 (71.4%) |  | 40 (54.1%) |
| high  (800 mg/30 min + 960 mg/120 min) |  | 46 (33.6%) |  | 30 (32.3%) |  | 16 (36.4%) |  | 17 (27.0%) |  | 29 (39.2%) |
| other |  | 6 (4.4%) |  | 2 (2.2%) |  | 4 (9.1%) |  | 1 (1.6%) |  | 5 (6.8%) |
| Additional therapy, no. (%) | 137 |  | 93 |  | 44 |  | 63 |  | 74 |  |
| prothrombin complex concentrate |  | 5 (3.6%) |  | 2 (2.2%) |  | 3 (6.8%) |  | 2 (3.2%) |  | 3 (4.1%) |
| tranexamic acid |  | 6 (4.4%) |  | 3 (3.2%) |  | 3 (6.8%) |  | 2 (3.2%) |  | 4 (5.4%) |
| ICH evacuation surgery |  | 15 (10.9%) |  | 1 (1.1%) |  | 14 (31.8%) |  | 0 (0.0%) |  | 15 (20.3%) |
| Concomitant medication, no (%) | 137 |  | 93 |  | 44 |  | 63 |  | 74 |  |
| antiplatelet therapy |  | 10 (7.3%) |  | 6 (6.5%) |  | 4 (9.1%) |  | 4 (6.3%) |  | 6 (8.1%) |
| Acetylsalicylic acid |  | 7 (5.1%) |  | 4 (4.3%) |  | 3 (6.8%) |  | 4 (6.3%) |  | 3 (4.1%) |
| Clopidogrel |  | 3 (2.2%) |  | 2 (2.2%) |  | 1 (2.3%) |  | 0 (0.0%) |  | 3 (4.1%) |
| nonsteroidal anti-inflammatory drugs |  | 3 (2.2%) |  | 1 (1.1%) |  | 2 (4.5%) |  | 1 (1.6%) |  | 2 (2.7%) |
| Diclofenac |  | 2 (1.5%) |  | 1 (1.1 %) |  | 1 (2.3%) |  | 1 (1.6%) |  | 1 (1.4%) |
| Ibuprofen |  | 1 (0.7%) |  | 0 (0.0%) |  | 1 (2.3%) |  | 0 (0.0%) |  | 1 (1.4%) |
| Medical history, no (%) | 137 |  | 93 |  | 44 |  | 63 |  | 74 |  |
| atrial fibrillation |  | 121 (88.3%) |  | 83 (89.2%) |  | 38 (86.4%) |  | 58 (92.1%) |  | 63 (85.1%) |
| cancer / brain metastases |  | 19 (13.9%) |  | 13 (14.0%) |  | 6 (13.6%) |  | 10 (15.9%) |  | 9 (12.2%) |
| coronary artery disease |  | 23 (16.8%) |  | 14 (15.1%) |  | 9 (20.5%) |  | 10 (15.9%) |  | 13 (17.6%) |
| deep vein thrombosis |  | 9 (6.6%) |  | 5 (5.4%) |  | 4 (9.1%) |  | 3 (4.8%) |  | 6 (8.1%) |
| diabetes |  | 32 (23.4%) |  | 23 (24.7%) |  | 9 (20.5%) |  | 15 (23.8%) |  | 17 (23.0%) |
| heart failure |  | 27 (19.7%) |  | 16 (17.2%) |  | 11 (25.0%) |  | 12 (19.0%) |  | 15 (20.3%) |
| hyperlipidemia |  | 39 (28.5%) |  | 31 (33.3%) |  | 8 (18.2%) |  | 22 (34.9%) |  | 17 (23.0%) |
| hypertension |  | 110 (80.3%) |  | 77 (82.8%) |  | 33 (75.0%) |  | 57 (90.5%) |  | 53 (71.6%) |
| intracranial bleeding |  | 12 (8.8%) |  | 9 (9.7%) |  | 3 (6.8%) |  | 6 (9.5%) |  | 6 (8.1%) |
| myocardial infarction |  | 10 (7.3%) |  | 5 (5.4%) |  | 5 (11.4%) |  | 5 (7.9%) |  | 5 (6.8%) |
| peripheral arterial occlusive disease |  | 9 (6.6%) |  | 7 (7.5%) |  | 2 (4.5%) |  | 5 (7.9%) |  | 4 (5.4%) |
| pulmonary embolism |  | 9 (6.6%) |  | 5 (5.4%) |  | 4 (9.1%) |  | 3 (4.8%) |  | 6 (8.1%) |
| stroke |  | 21 (15.3%) |  | 17 (18.3%) |  | 4 (9.1%) |  | 12 (19.0%) |  | 9 (12.2%) |
| transient ischemic attack |  | 7 (5.1%) |  | 5 (5.4%) |  | 2 (4.5%) |  | 4 (6.3%) |  | 3 (4.1%) |

NIHSS: National Institutes of Health Stroke Scale; mRS: modified Rankin Scale.


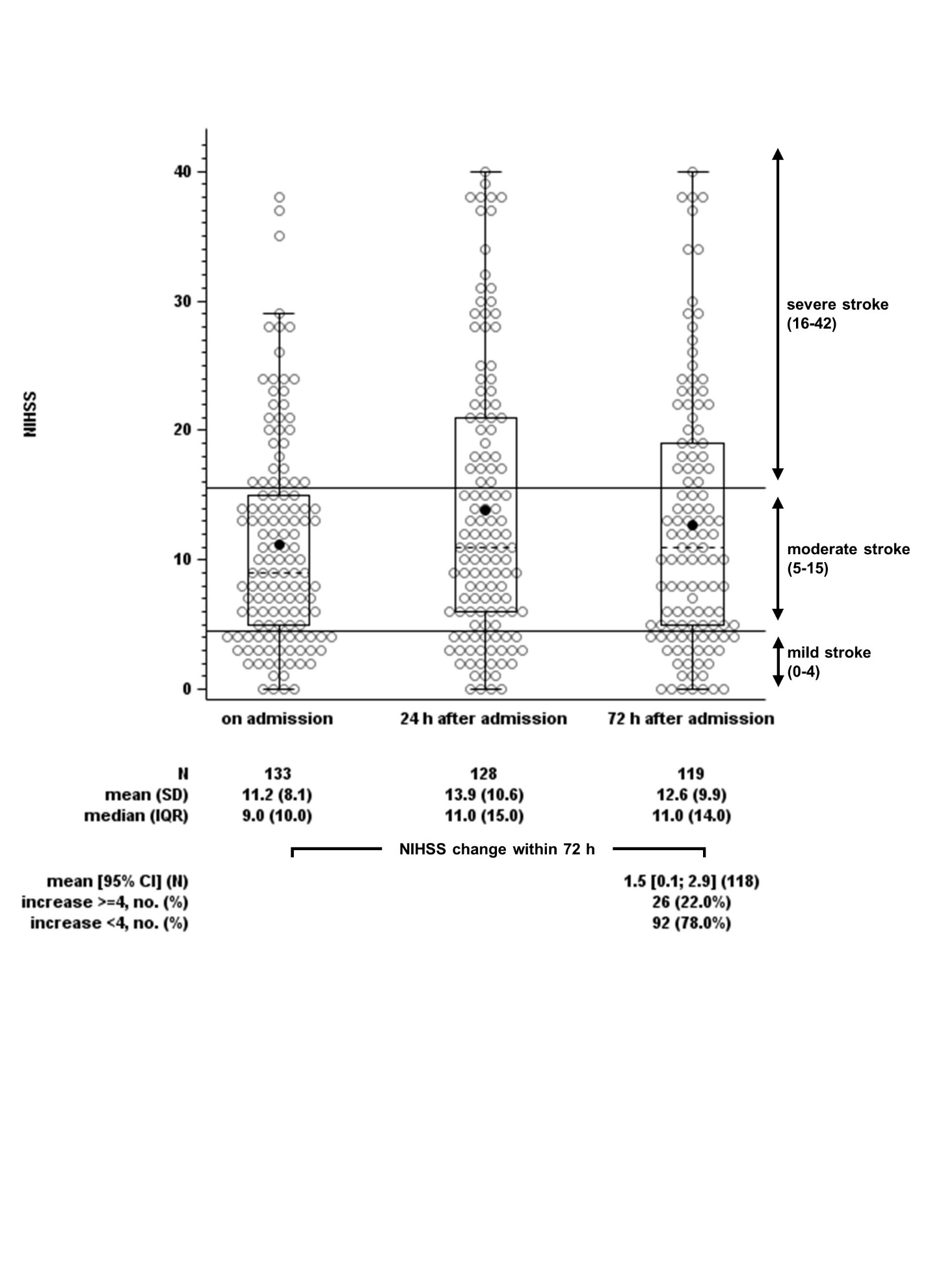


**Figure S1.** Stroke severity according to the National Institutes of Health Stroke Scale (NIHSS) shown as boxplots (box and tukey whiskers).


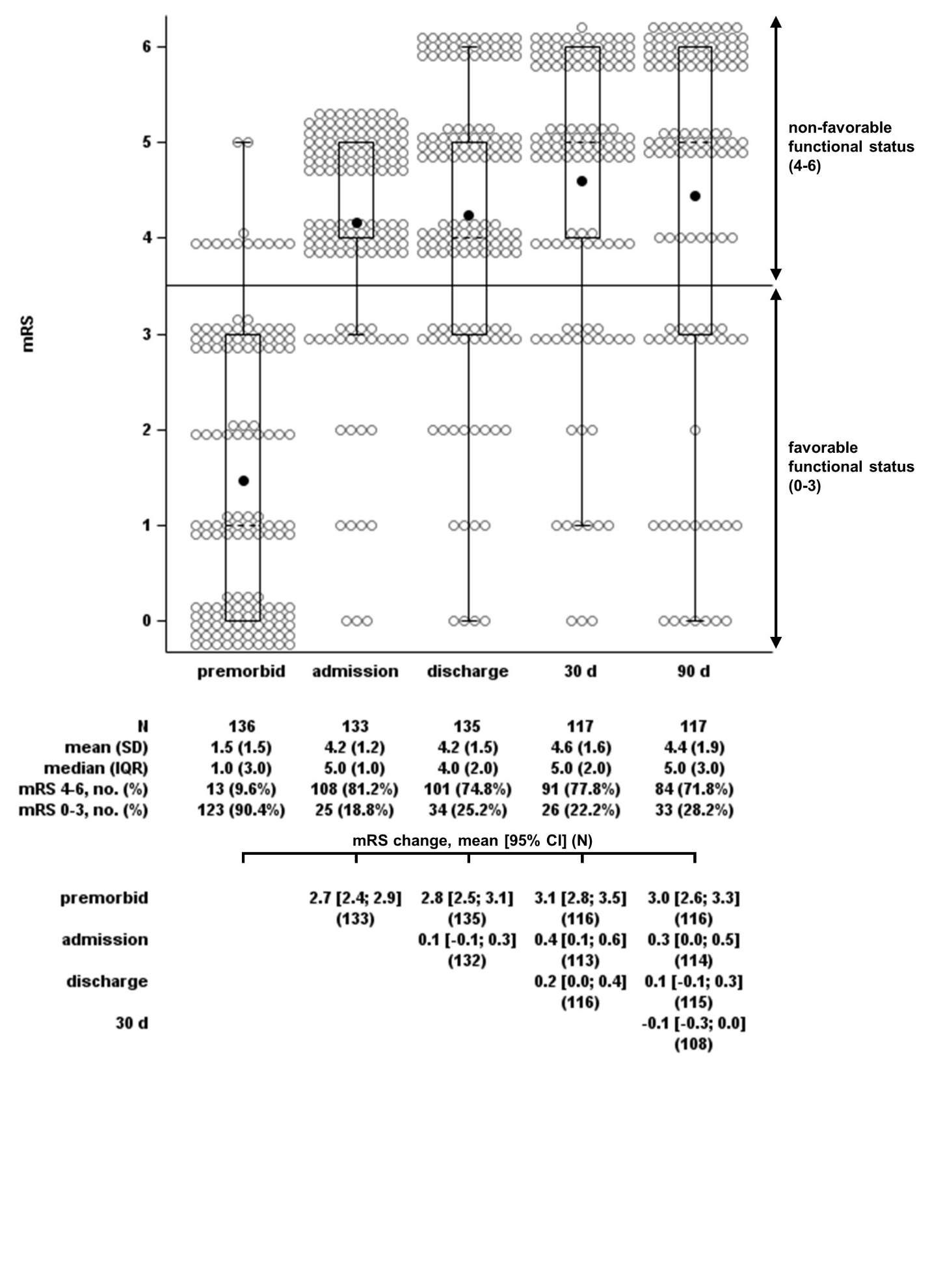


**Figure S2.** Functional status according to the modified Rankin Scale (mRS) shown as boxplots (box and tukey whiskers).


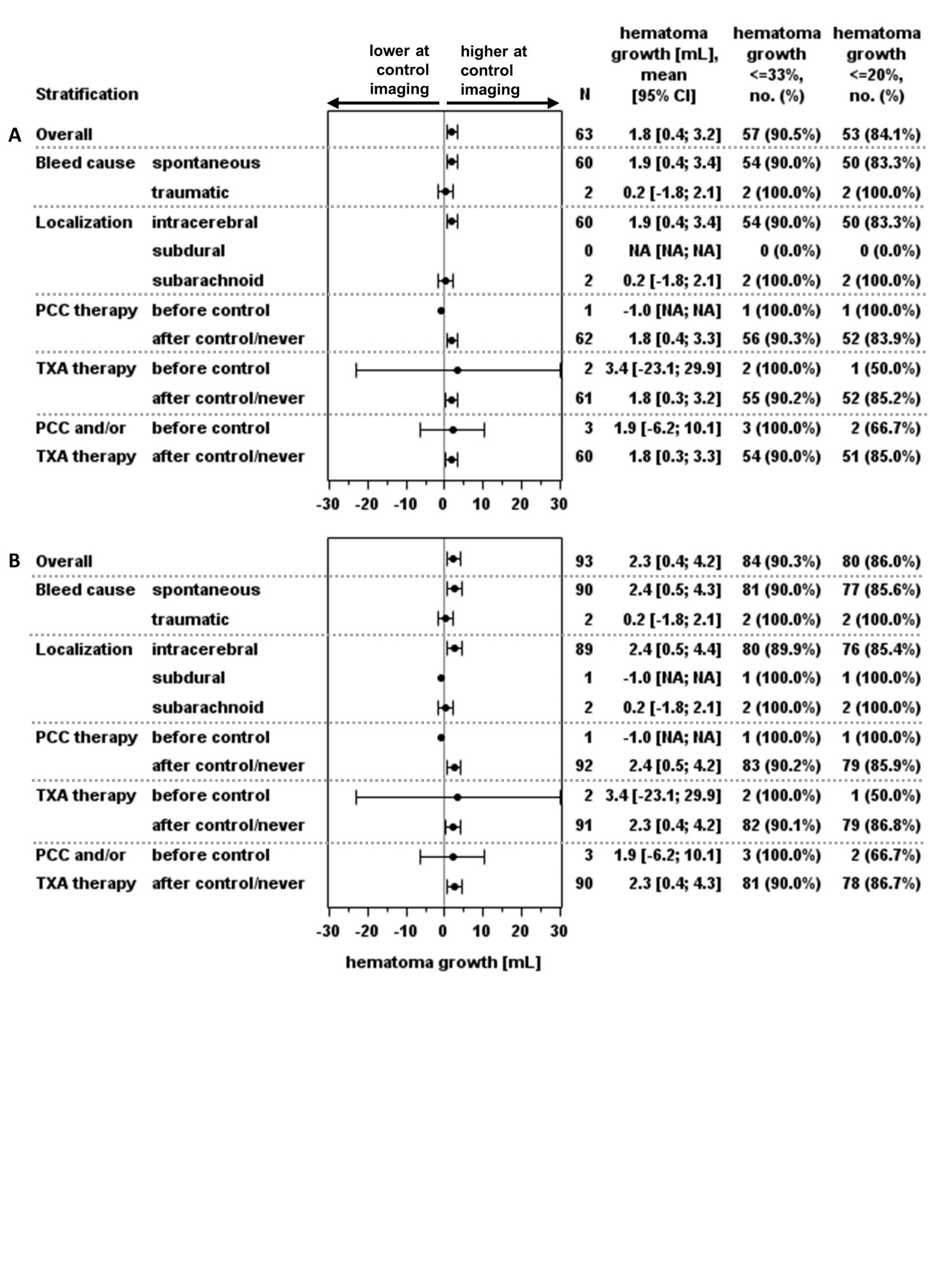


**Figure S3.** Further subgroup analyses of mean hematoma volume change within 12-72 h (**A**) and until first control (**B**). PCC: prothrombin complex concentrate; TXA: tranexamic acid.

**ASTRO-DE Investigators Group**

| Marcus Ohlrich  Jens Schaumberg | Neurologie, Sana Kliniken Lübeck, Lübeck, Germany |
| --- | --- |
| Matthias Maschke | Department of Neurology, Campus Trier, University of Mainz, Trier, Germany |
| Georg Royl | Department of Neurology, University of Lübeck, Lübeck, Germany |
| Dominik Michalski | Department of Neurology, University of Leipzig, Leipzig, Germany |
| Peter Kraft | Klinikum Main-Spessart, Lohr, Germany |
| Hassan Soda  Renate Weinhardt | Klinik für Akutneurologie, RHÖN-KLINIKUM Campus Bad Neustadt, Bad Neustadt an der Saale, Germany |
| Corina Epple | Klinik für Neurologie, Klinikum Hanau, Hanau, Germany |
| Arno Reich | Klinik für Neurologie, Uniklinik RWTH Aachen, Aachen, Germany |
| Thorsten Steiner | Klinik für Neurologie, Klinikum Frankfurt Höchst, Frankfurt am Main, Germany  Klinik für Neurologie, Universitätsklinik Heidelberg, Heidelberg, Germany |
| Katharina Althaus | Neurologische Universitätsklinik, Universitäts- und Rehabilitationskliniken Ulm, Ulm, Germany |
| Paul Sparenberg | Klinik für Neurologie, BG Klinikum Unfallkrankenhaus Berlin, Berlin, Germany |
| Jan-Hendrik Schäfer | Klinik für Neurologie, Universitätsklinikum Frankfurt, Frankfurt am Main, Germany |
| Christian Urbanek | Neurologische Klinik mit Klinischer Neurophysiologie, Klinikum Ludwigshafen, Ludwigshafen, Germany |
| Matthias Wittstock | Klinik und Poliklinik für Neurologie, Universitätsmedizin Rostock, Rostock, Germany |
| Frank Trostdorf  Florian Ehlers | Klinik für Neurologie, Agaplesion Bethesda Krankenhaus Bergedorf, Hamburg, Germany |
| Peter Arthur Ringleb | Klinik für Neurologie, Universitätsklinikum Heidelberg, Heidelberg, Germany |
| Martin Nückel | Klinik für Neurologie, Universitätsklinik der Paracelsus Medizinischen Privatuniversität, Nürnberg, Germany |
| Bernd Kallmünzer | Klinik für Neurologie, Uniklinikum Erlangen, Erlangen, Germany |
| Tobias Neumann-Haefelin  Jörg Berthel | Klinik für Neurologie, Campus Fulda, Universitätsklinikum Marburg, Fulda, Germany |
| Andreas Dietz | Klinik für Neurologie, Hochtaunusklinik Bad Homburg, Bad Homburg, Germany |

**STROBE Statement – checklist of items that should be included in reports of observational studies**

|  | **Item No.** | **Recommendation** | | **Page  No.** | **Relevant text from manuscript** |  |
| --- | --- | --- | --- | --- | --- | --- |
| **Title and abstract** | 1 | (*a*) Indicate the study’s design with a commonly used term in the title or the abstract. | | 1  2 | line 1-2 (title)  line 52-53 (abstract) |  |
|  |  | (*b*) Provide in the abstract an informative and balanced summary of what was done and what was found. | | 2, 3 | line 42-76 |  |
| **Introduction** | | | | |  |  |
| Background /  rationale | 2 | Explain the scientific background and rationale for the investigation being reported. | | 4 | line 77-95 |  |
| Objectives | 3 | State specific objectives, including any prespecified hypotheses. | | 4 | line 96-98 |  |
| **Methods** | | | | |  |  |
| Study design | 4 | Present key elements of study design early in the paper. | | 5 | line 102-104 |  |
| Setting | 5 | Describe the setting, locations, and relevant dates, including periods of recruitment, exposure, follow-up, and data collection. | | 5  5 | line 102  line 104-120 |  |
| Participants | 6 | (*a*) *Cohort study*—Give the eligibility criteria, and the sources and methods of selection of participants. Describe methods of follow-up. | | 5-6  5 | line 121-126  line 113-116 |  |
|  |  | (*b*) *Cohort study*—For matched studies, give matching criteria and number of exposed and unexposed. | | NA | NA |  |
| Variables | 7 | Clearly define all outcomes, exposures, predictors, potential confounders, and effect modifiers. Give diagnostic criteria, if applicable. | | 6 | line 127-136 |  |
| Data sources/ measurement | 8* | For each variable of interest, give sources of data and details of methods of assessment (measurement). Describe comparability of assessment methods if there is more than one group. | | 5  6 | line 105-120  line 122-126 |  |
| Bias | 9 | Describe any efforts to address potential sources of bias. | | 6  7 | line 128-130  line 147-150 |  |
| Study size | 10 | Explain how the study size was arrived at. | | 6 | line 138-143 |  |
| Quantitative variables | 11 | Explain how quantitative variables were handled in the analyses. If applicable, describe which groupings were chosen and why. | | 6  6-7 | line 128-136  line 143-145 |  |
| Statistical methods | 12 | (*a*) Describe all statistical methods, including those used to control for confounding. | | 6-7 | line 137-153 |  |
|  |  | (*b*) Describe any methods used to examine subgroups and interactions. | | 6  7 | line 128-130  line 147-150 |  |
|  |  | (*c*) Explain how missing data were addressed. | | 7 | line 145-147 |  |
|  |  | (*d*) *Cohort study*—If applicable, explain how loss to follow-up was addressed. | | 5 | line 113-116 |  |
|  |  | (*e*) Describe any sensitivity analyses. | | NA | NA |  |
| **Results** | | | | | |  |
| Participants | 13* | (a) Report numbers of individuals at each stage of study – e.g. numbers potentially eligible, examined for eligibility, confirmed eligible, included in the study, completing follow-up, and analyzed. | | 7  19 | line 157-158  figure 1 |  |
|  |  | (b) Give reasons for non-participation at each stage. | | 19 | figure 1 |  |
|  |  | (c) Consider use of a flow diagram. | | 19 | figure 1 |  |
| Descriptive data | 14* | (a) Give characteristics of study participants (e.g. demographic, clinical, social) and information on exposures and potential confounders. | | 7, 8  8  9  17  21  Suppl.  Suppl. | line 158-172  line 174-179  line 191-196  table 1  figure 3A  table S1  figure S1-S2 |  |
|  |  | (b) Indicate number of participants with missing data for each variable of interest. | | 17  21  Suppl.  Suppl. | table 1  figure 3A  table S1  figure S1-S2 |  |
|  |  | (c) *Cohort study*—summarize follow-up time (e.g., average and total amount). | | 18  19 | table 2  figure 1 |  |
| Outcome data | 15* | *Cohort study*—Report numbers of outcome events or summary measures over time. | | 8  8, 9  17, 18  20-22  Suppl.  Suppl. | line 172-181  line 185-196  table 1-2  figure 2-4  table S2-S8  figure S1-S3 |  |
| Main results | 16 | (*a*) Give unadjusted estimates and, if applicable, confounder-adjusted estimates and their precision (e.g., 95% confidence interval). Make clear which confounders were adjusted for and why they were included. | | 8  8-9  18  20-22  Suppl.  Suppl. | line 177-186  line 188-209  table 2  figure 2-4  table S2-S8  figure S1-S3 |  |
|  |  | (*b*) Report category boundaries when continuous variables were categorized. | | 18  22 | table 2  figure 4 |  |
|  |  | (*c*) If relevant, consider translating estimates of relative risk into absolute risk for a meaningful time period. | | NA | NA |  |
| Other analyses | 17 | | Report other analyses done – e.g. analyses of subgroups and interactions, and sensitivity analyses. | 8  8  18  21  22  Suppl. Suppl. | line 175-179  line 184-186  table 2  figure 3A  figure 4  table S2-S4, S7-S8  figure S3 | |
| **Discussion** | | | | | |  |
| Key results | 18 | | Summarize key results with reference to study objectives. | 9-13 | line 211-288 | |
| Limitations | 19 | | Discuss limitations of the study, taking into account sources of potential bias or imprecision. Discuss both direction and magnitude of any potential bias. | 10, 11  11, 12  12  13 | line 220-237  line 245-271  line 278-279  line 284-288 | |
| Interpretation | 20 | | Give a cautious overall interpretation of results considering objectives, limitations, multiplicity of analyses, results from similar studies, and other relevant evidence. | 13  13 | line 280-288  line 291-295 | |
| Generalizability | 21 | | Discuss the generalizability (external validity) of the study results. | 9-13 | line 211-288 | |
| **Other information** | | |  | | |  |
| Funding | 22 | | Give the source of funding and the role of the funders for the present study and, if applicable, for the original study on which the present article is based. | 15 | line 334-338 | |

*Give information separately for cases and controls in case-control studies and, if applicable, for exposed and unexposed groups in cohort and cross-sectional studies.

**Note:** An Explanation and Elaboration article discusses each checklist item and gives methodological background and published examples of transparent reporting. The STROBE checklist is best used in conjunction with this article (freely available on the Web sites of PLoS Medicine at http://www.plosmedicine.org/, Annals of Internal Medicine at http://www.annals.org/, and Epidemiology at http://www.epidem.com/). Information on the STROBE Initiative is available at www.strobe-statement.org.
